# Phenotypic Diversity of Cardiomyopathy Caused by an *MYBPC3* Frameshift Mutation in a Korean Family: A Case Report

**DOI:** 10.3390/medicina57030281

**Published:** 2021-03-18

**Authors:** Joonhong Park, Jong-Min Lee, Jung Sun Cho

**Affiliations:** 1 Department of Laboratory Medicine, Jeonbuk National University Medical School and Hospital, Jeonju 54907, Korea; miziro@jbnu.ac.kr; 2 Research Institute of Clinical Medicine of Jeonbuk National University-Biomedical Research Institute of Jeonbuk National University Hospital, Jeonju 54907, Korea; 3 Department of Cardiology, College of Medicine, The Catholic University of Korea, Seoul 06591, Korea; eco_sirius@naver.com

**Keywords:** restrictive cardiomyopathy, hypertrophic cardiomyopathy, phenotypic diversity, cardiac magnetic resonance image, *MYBPC3* mutation, whole exome sequencing

## Abstract

Restrictive cardiomyopathy (RCM) is one of the rarest cardiac disorders, with a very poor prognosis, and heart transplantation is the only long-term treatment of choice. We reported that a Korean family presented different cardiomyopathies, such as idiopathic RCM and hypertrophic cardiomyopathy (HCM), caused by the same *MYBPC3* mutation in different individuals. A 74-year-old male was admitted for the evaluation of exertional dyspnea, palpitations, and pitting edema in both legs for several months. Transthoracic echocardiography (TTE) showed RCM with biatrial enlargement and pericardial effusion. Cardiac magnetic resonance (CMR) images revealed normal left ventricular chamber size, borderline diffuse left ventricular hypertrophy and very large atria. In contrast to the proband, CMR images showed asymmetric septal hypertrophy of the left ventricle, consistent with a diagnosis of HCM in the proband’s two daughters. Of the five heterozygous variants identified as candidate causes of inherited cardiomyopathy by whole exome sequencing in the proband, Sanger sequencing confirmed the presence of a heterozygous frameshift mutation (NM_000256.3:c.3313_3314insGG; p.Ala1105Glyfs*85) in *MYBPC3* in the proband and his affected daughters, but not in his unaffected granddaughter. There is clinical and genetic overlap of HCM with restrictive physiology and RCM, especially when HCM is combined with severe myocardial fibrosis. Family screening with genetic testing and CMR imaging could be excellent tools for the evaluation of idiopathic RCM.

## 1. Introduction

Restrictive cardiomyopathy (RCM) is one of the rarest cardiac disorders, with a very poor prognosis, and heart transplantation is the only long-term treatment of choice; RCM is less common than dilated cardiomyopathy (DCM) and hypertrophic cardiomyopathy (HCM) [1]. Most cases of RCM have secondary causes, such as inflammatory, infiltrative, or systemic disease [2]. Genetic RCM, especially in patients with familial RCM, has been suggested to have a genetic spectrum [1]. Sarcomeric and cytoskeletal gene mutations and sarcomere protein mutations have been reported to be related to idiopathic RCM, similar to mutations in genes such as *CRYAB* [3], *DES* [4], *FLNC* [5], *LAMP2*, *LMNA*, *MYH7* [6], *MYBPC3* [7], *TNNI3*, *TNNT2*, and *TPN1* identified in HCM and DCM, but in total, the mutation detection rate is quite low, corresponding to approximately 30% [1]. However, many genes have been reported to be related to other cardiomyopathies, and most cardiomyopathies share the same genotype groups. Significantly, the mechanisms underlying the phenotypic diversity of cardiomyopathy caused by mutations in the same genes and the basis for the variable expressivity of cardiomyopathy phenotypes within family members are poorly understood [8,9]. To date, substantial advances in the understanding of the genetic causes of cardiomyopathies have become feasible because of the development of high-throughput approaches to DNA sequencing, namely, massively parallel sequencing (MPS) [10,11]. Furthermore, restrictive ventricular physiology, which is known to be one of the distinct hemodynamic characteristics of RCM, can be observed in HCM [12]. Therefore, there could be clinical and etiological overlap between RCM and HCM with restrictive physiology.

In this report, we described a case of phenotypic diversity of familial cardiomyopathy, such as idiopathic RCM and HCM, caused by the same *MYBPC3* mutation in a Korean family.

## 2. Case Presentation

**Figure 1:**
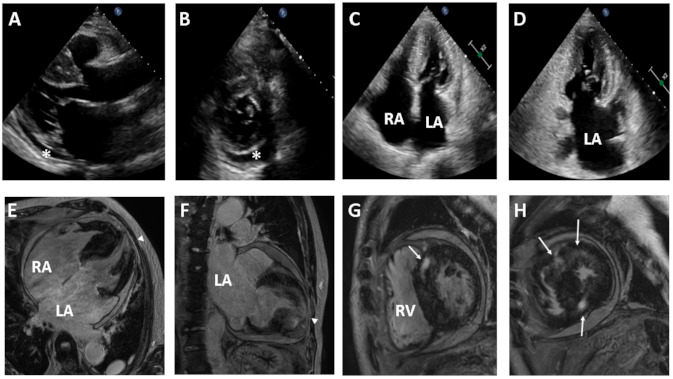
Transthoracic echocardiography (TTE) (upper panel) and contrast-enhanced cardiac magnetic resonance (CMR) images (lower panel) from the proband with idiopathic restrictive cardiomyopathy. Two-dimensional TTE reveals a normal left ventricle chamber (**A**), small pericardial effusion (asterisk) (**B**), and biatrial enlargement (**C**,**D**). CMR images demonstrate biatrial enlargement and transmural late gadolinium enhancement (LGE) in the apical wall (arrowhead) (**E**,**F**) and mid myocardial patchy LGE in the ventricular septum (arrow) (**G**,**H**). LA, left atrium; RA, right atrium; RV, right ventricle.

A 74-year-old male was admitted to the Department of Cardiology, Daejeon St. Mary’s Hospital (Daejeon, Korea), for the evaluation of exertional dyspnea (New York Heart Association (NYHA) functional class III), palpitations, and bilateral leg pitting edema for several months. His electrocardiography and Holter monitoring showed atrial fibrillation and rare premature ventricular complexes. He underwent transthoracic echocardiography (TTE), which showed RCM with biatrial enlargement and small pericardial effusion (Figure 1A–D). His coronary artery angiogram was normal and other secondary causes of RCM were not found. Cardiac magnetic resonance (CMR) images revealed normal left ventricle (LV) chamber size, borderline diffuse left ventricular hypertrophy and very large atria. Contrast enhanced CMR images demonstrated extensive transmural late gadolinium enhancement (LGE) in the apical wall and mid myocardial patchy LGE in the ventricular septum (Figure 1E–H).

**Figure 2:**
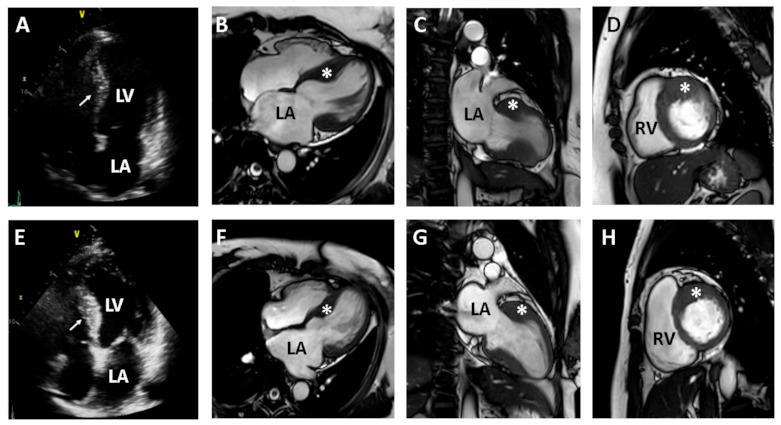
Two-dimensional echocardiography and contrast-enhanced cardiac magnetic resonance (CMR) images from the proband’s daughters with hypertrophic cardiomyopathy (HCM) (second daughter in upper panel and fourth daughter in lower panel). Two-dimensional echocardiography reveals the hypertrophy of the ventricular septum in each daughter ((**A**) in second daughter and (**E**) in fourth daughter). CMR images clearly demonstrate segmental hypertrophy (asterisk) confined to the ventricular septum, consistent with a diagnosis of HCM ((**B**–**D**) in second daughter and (**F**–**H**) in fourth daughter). LA, left atrium; RA, right atrium; LV, left ventricle; RV, right ventricle.

We recommended clinical evaluation and genetic counseling for his family members because he presented a family history of heart disease, with two affected daughters. The proband’s second daughter had mild exertional chest discomfort. Her TTE showed asymmetric septal hypertrophy of the LV and a mildly dilated left atrium (LA) (Figure 2A–D). Her diastolic dysfunction was grade I according to the American Society of Echocardiography and the European Association of Cardiovascular Imaging criteria [13]. The proband’s fourth daughter also had asymmetric septal hypertrophy of the LV, with mild enlargement of the LA (Figure 2E–H). CMR images of the two daughters showed asymmetric septal hypertrophy of the LV, consistent with a diagnosis of HCM. LGE was not found in either daughter.

**Figure 3:**
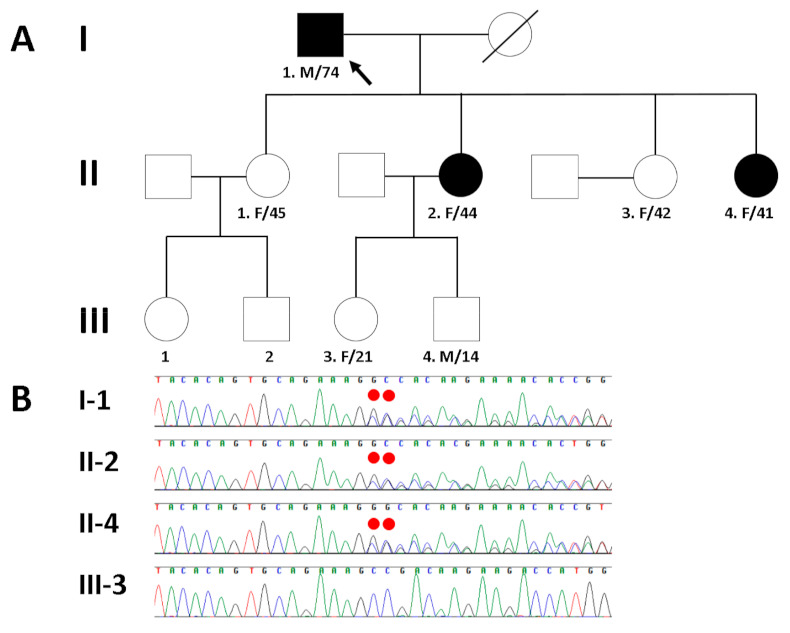
(**A**) Pedigree of the proband (arrow) and his family members with different cardiomyopathies caused by a heterozygous *MYBPC3* frameshift mutation. (**B**) Sanger sequencing confirmed a heterozygous frameshift mutation (NM_000256.3:c.3313_3314insGG; p.Ala1105Glyfs*85) of *MYBPC3*, which was inherited in an autosomal dominant manner in the proband (I-1) and his affected daughters (II-2 and II-4), but not in his unaffected granddaughter (III-3). The mutation is highlighted by the red dots.

To resolve the potential genetic cause of cardiomyopathy in a Korean family (I-1, II-2, and II-4 in Figure 3A), the proband’s genomic DNA was analyzed by whole exome sequencing (WES). The exomic DNA of the proband was enriched using Agilent’s SureSelect XT Human All Exon v5 (Agilent Technologies, Santa Clara, CA, USA), and paired-end sequencing was conducted on an Illumina HiSeq2500 (Illumina, San Diego, CA, USA) for the detection of variants, given the suspicion of familial disease. Base calling, alignment, variant calling, annotation, and quality control reporting were performed using a GATK Best Practices workflow for germline short variant discovery and were manually reviewed by medical laboratory geneticists. In particular, the annotation of the identified variants with respect to the results of their mutation in the reported genes was estimated using the dbNSFP [14] and Ensembl Variant Effect Predictor [15]. By estimating sequence quality along all sequences, 5973 million reads were generated from the proband. A percentage of bases above the average of 30× was achieved for 92.3% of the target region, and the mean read depth (×) was 110. WES of the proband identified five heterozygous variants within the coding region of each gene as candidate causes of autosomal dominant inherited heart diseases with a population allele frequency within the Genome Aggregation Database < 0.001. To determine which variant was the cause of cardiomyopathy in the affected family members, Sanger sequencing was performed additionally. As a result, Sanger sequencing confirmed the presence of a heterozygous frameshift mutation (NM_000256.3:c.3313_3314insGG; p.Ala1105Glyfs*85) in *MYBPC3* in the proband (I-1) and his affected daughters (second (II-2) and fourth (II-4)), but not in his unaffected granddaughter (III-3) (Figure 3B). The variant is not listed in public genome databases from gnomAD (https://gnomad.broadinstitute.org/; accessed on 8 November 2020) and the Korean Reference Genome Database (http://coda.nih.go.kr/coda/KRGDB/index.jsp; accessed on 8 November 2020). Although the four remaining variants (*KALRN*: NM_007064.5: c.1702C>T, p.Pro568Ser; *KCNH2*: NM_000238.4:c.442C>T, p.Arg148Trp; *SKI*: NM_003036.4: c.30T>G, p.Cys10Trp; *TTN*: NM_133378.4: c.54293T>C, p.Ile18098Thr) were the likely candidates, there was no exact correlation between the genotype of these variants and the clinical phenotype in the proband and his family members as determined by Sanger sequencing. Therefore, the *MYBPC3* frameshift mutation seemed to be the pathogenic variant.

## 3. Discussion

The genetic spectrum of RCM using an MPS approach reported that idiopathic RCM is primarily a genetic disease [1,10]. Several variants in genes encoding ion channels and desmosomal proteins in patients with RCM were identified in 50% to 60% of RCM patients [1,16]. Similarly, of more than 1500 mutations in at least 11 genes related to HCM, *MYBPC3* is the most frequently mutated gene, resulting in 30% to 40% of all cases of HCM [17]. However, *MYBPC3* mutations in patients with HCM [18] and DCM [19] were reported in several studies, and a few mutations were described previously as disease-causing in RCM [1,10]. Here, we report a Korean family presenting different cardiomyopathies, such as idiopathic RCM and HCM, caused by the same *MYBPC3* mutation in different individuals (Table 1). In this case, the proband also complained of severe dyspnea refractory to heart failure medication and suffered from bilateral leg pitting edema, pulmonary venous congestion and atrial fibrillation. Particularly in RCM, male sex, age older than 70 years, high NYHA class and left atrial dimension >60 mm were associated with poor prognosis [2,12]. Severe diastolic dysfunction and restrictive filling with elevated filling pressures, as one of the distinct characteristics of RCM, could also be observed in HCM. Therefore, in patients who fulfill the diagnostic criteria for RCM without evidence of secondary causes, familial evaluation to exclude HCM is recommended.

The proband’s two daughters had familial HCM, which suggested that idiopathic RCM might be a different phenotype than familial HCM. Similar to the phenotype of the proband’s two daughters, this frameshift mutation has been previously reported to be related to Korean HCM [11]. An intriguing aspect of sarcomere protein mutations is pleiotropy. Accordingly, mutations in the same gene could manifest as HCM, RCM, DCM, and even left ventricular non-compaction syndrome. It may reflect the location of the mutations in different domains of the protein, leading to differential interactions of the mutant proteins with the other protein constituents of sarcomeres and activating different sets of intermediary molecular events [20]. The variability in the phenotypic expression of HCM is in part due to the effects of modifier genetic variants and environmental factors. The modifier variants, unlike the causal mutations, simply influence the expression of HCM, each exerting a modest effect. In accord with the diversity of the human genome, the modifier variants are also expected to differ among individuals and hence, in part, explain inter-individual variability in the phenotypic expression of HCM [20]. Therefore, first degree family members of patients with HCM should be evaluated for possible inheritance of the disease, including a medical history, physical examination, electrocardiography, and echocardiography. Routine genetic screening of first degree relatives is not recommended unless a definite HCM-causing mutation has been identified in the index case. The findings of this case suggested that not only HCM but also idiopathic RCM need to be evaluated in family screening. The clinical characteristics and the penetrance and phenotypic burden of HCM in individuals with a founder *MYBPC3* mutation were reported [21,22]. In the Dutch population, the clinical phenotype and outcome of HCM with a founder *MYBPC3* mutation (c.2373dupG, c.2827C>T or c.2864_2865delCT) were similar to those of HCM with a non-founder *MYBPC3* mutation, but worse than those of wildtype HCM [21]. In the Icelandic population, the penetrance of HCM was influenced by age and sex. Specifically, left ventricular hypertrophy with a founder *MYBPC3* mutation (c.927-2A>G) was present in 39% of males but only 9% of females under the age of 40 years (*p* = 0.015), versus 86% and 83%, respectively, after the age of 60 (*p* = 0.89) [22]. Similarly, echocardiography of the proband’s two daughters showed mild septal hypertrophy (18 mm and 17 mm). However, CMR images clearly demonstrated reverse septal curvature hypertrophy, consistent with a diagnosis of HCM [23,24]. CMR imaging and genetic testing have increased the recognition of the HCM phenotype and improved clinical diagnosis [25,26]. CMR imaging of the proband showed extensive LGE in the apical area of the LV. Advanced myocardial fibrosis in patients with end-stage HCM could provoke the atrophy of the myocardium, which could be the cause of the relatively normal size of the LV chamber of probands with a predominantly restrictive CMR phenotype [27].

## 4. Conclusions

In conclusion, we described a case of phenotypic diversity of familial cardiomyopathy, such as idiopathic RCM and HCM, caused by the same *MYBPC3* mutation. There is clinical and genetic overlap of HCM with restrictive physiology and RCM, especially when HCM is combined with severe myocardial fibrosis. Our findings further underline the genetic and pathophysiological link between HCM and RCM. Family screening with genetic tests and CMR images could be excellent tools for the evaluation of idiopathic RCM.

## Figures and Tables

**Table 1:** Clinical characteristics of individuals diagnosed as different cardiomyopathy with heterozygous *MYBPC3* frameshift mutation.

Characteristics	Proband (I-1)	Second Daughter (II-2)	Fourth Daughter (II-4)
Diagnosis	Restrictive cardiomyopathy	Hypertrophic cardiomyopathy	Hypertrophic cardiomyopathy
Sex/Age(year)	Male/74	Female/44	Female/41
Electrocardiography and Holter monitoring	Atrial fibrillation and rare premature ventricular contractions	Sinus rhythm with frequent premature ventricular contractions	Sinus rhythm with rare premature atrial contractions and premature ventricular contractions
Transthoracic echocardiography	Biatrial enlargement and pericardial effusion	Asymmetric septal hypertrophy of LV and mildly dilated LA	Asymmetric septal hypertrophy of LV with mild LA enlargement
	LVEDD 50 mm	LVEDD 40 mm	LVEDD 39 mm
	IVS 18 mm	IVS 17 mm	IVS 18.4 mm
	EF 60.2%	EF 64.2%	EF 64.2%
	E/e’ 14.1	E/e’ 9	E/e’ 10.1
	e’ 5.2 cm/s	e’ 9.9 cm/s	e’ 7.5 cm/s
	TR peak velocity 3.8 m/s	TR peak velocity 1.8 m/s	TR peak velocity 2.5 m/s
	LAVI 115 mL/m^2^	LAVI 41 mL/m^2^	LAVI 40.1 mL/m^2^

LVEDD, left ventricular end-diastolic dimension; IVS, interventricular septal dimension; EF, ejection fraction; E/e’, the ratio of early transmitral flow velocity to early diastolic velocity of the mitral annulus; e’, septal tissue velocity; TR, tricuspid regurgitation; LAVI, left atrial volume index; LV, left ventricle; LA, left atrium.

## Data Availability

The data presented in this study are available on request from the corresponding author.
